# Wicked Questions and Perfect Storms: Comment on "From Local Action to Global Policy: A Comparative Policy Content Analysis of National Policies to Address Musculoskeletal Health to Inform Global Policy Development"

**DOI:** 10.34172/ijhpm.8620

**Published:** 2024-12-18

**Authors:** Faye Deane

**Affiliations:** School of Health and Life Sciences, Teesside University, Middlesbrough, UK.

**Keywords:** Musculoskeletal, Health Systems, Policy, Integrated Care

## Abstract

Schneider and colleagues’^1^ paper "From Local Action to Global Policy: A Comparative Policy Content Analysis of National Policies to Address Musculoskeletal Health to Inform Global Policy Development" examines musculoskeletal (MSK) policies across 30 of the world’s most populous nations using an adapted World Health Organization (WHO) Health Systems Building Blocks (HSBB) framework. This commentary critiques the findings through a complexity theory lens, emphasizing the amplified wickedness of MSK health challenges, shaped by systemic, socioeconomic, situational, and medical factors. These interconnected elements create varying levels of complexity, making policy implementation increasingly difficult. The commentary calls for addressing funding gaps, promoting integrated care models, and adopting a life-course approach to align MSK policy with global priorities like healthy aging. It advocates for shifting from policy learning to culturally sensitive, actionable implementation, ensuring that MSK healthcare can be effectively delivered in diverse, complex health ecosystems. This approach is critical for fostering equitable and sustainable MSK health solutions globally.

## Introduction

###  The Current Musculoskeletal Landscape

 Musculoskeletal disorders (MSDs) affect 1.71 billion people, significantly impairing quality of life.^2^ Over the past 30 years, MSD incidence has risen by 60%, driven by increased life expectancy, multimorbidity, sedentary lifestyles, and the growing prevalence of non-communicable diseases (NCDs).^3^ With cases projected to increase 115% by 2050, and the population aged 60 and over expected to triple, targeted policies are urgently needed.^4^ Despite this, Schneider and colleagues^1^ found that global policy development lags behind other NCDs, with limited integration into broader health frameworks and insufficient attention to socioeconomic disparities.

 As healthcare trends move toward integrated, cross-sector, collaborative care models, complexity increases. These systems, shaped by contextual differences, clinical cultures, and multi-agent environments, span micro-, meso-, and macro-level factors. As complexity increases, so does wickedness, placing musculoskeletal (MSK) healthcare systems in a “perfect storm,” of competing demands and diverse stakeholder needs. This complexity frames MSDs as a wicked problem requiring adaptive, nuanced solutions. Kupiers et al (as cited in Braithwaite et al^5^) describe how medical, situational, and systems complexities co-evolve into wicked problems, further compounding the challenges.

 While Schneider and colleagues’ reliance on the World Health Organization (WHO) Health Systems Building Blocks (HSBB) framework enables effective cross-country policy comparison, it falls short in addressing the dynamic and interconnected nature of health systems. By applying a complexity theory lens, this commentary draws on Complex Adaptive Systems (CAS), a framework that emphasizes adaptability, feedback loops, and interdependence. CAS offers a more robust approach to tackling the multifaceted challenges of MSD healthcare, addressing the wickedness inherent in these systems and guiding the development of more effective global policies.

###  Musculoskeletal Health Policies

 Schneider and colleagues’ analysis found only three countries employed system-wide, integrated approaches to comprehensively address MSDs, all from high-income nations, highlighting significant socio-economic disparity. Despite calls to prioritise global health system strengthening, MSDs have only been considered a priority since 2016,^6^ likely due to their perceived reversibility and low case fatality rate, despite waiting times for initial care often exceeding 20 weeks.^7,8^ Policies also fail to consider a life-course approach or to integrate MSK health into broader NCD frameworks, indicating a disconnect between policy and the wider MSK landscape.

 The analysis identified 47 sub-themes across 8 categories, with service delivery and workforce as key themes for global policy development. Few national policies addressed all the WHO HSBB framework’s policy themes, reflecting the varying developmental stages of MSK health systems. While the HSBB framework provides a systematic tool for cross-country comparisons, its reductionist design limits its ability to address MSD complexities. Criticized as a resource allocation tool rather than a research instrument,^9^ it overlooks critical factors like micro-level community care, meso-level regional integration, and macro-level governance.

 A complexity science approach could enhance the HSBB framework by offering a dynamic perspective on global MSK health policy, accounting for interactions among system agents, environmental factors, and performance.^10^ Demonstrating promise in implementation science, complexity theory explores system-wide interactions, highlighting how outcomes often arise unpredictably from the interconnections within a system.^5^ By emphasizing dynamic processes and the nonlinear nature of these interactions, it provides valuable insights into the challenges of achieving consistent, sustainable outcomes in complex environments. Simulation models and adaptive policy approaches, hallmarks of complexity theory, could enhance MSK interventions at macro-, micro-, and meso levels. However, while conceptually strong, complexity theory has yet to evolve into a fully integrated framework capable of addressing the diverse complexities of the MSK disease burden.^11^ Future research should focus on combining complexity-informed methods alongside traditional tools offering a more holistic approach to strengthening policy.

###  Musculoskeletal Disorder Health Policy 

####  Macro-Meso-Micro Level Mismatch

 The HSBB framework focuses on macro-structural dimensions in MSK health policy, with limited attention to meso- and micro-level factors. Schneider and colleagues^1^ emphasize the need to ground global policy in local experiences, but the framework lacks exploration of how micro-level processes, such as organizational dynamics, shape macro-level patterns and strategic direction. Additionally, it overlooks key population health factors, such as age and gender disparities. Incorporating localized, demand-sensitive solutions alongside macro-level policies would enhance the framework’s equity and applicability, ensuring it is more relevant and effective across diverse contexts.

 Notable meso-level MSK policy interventions including Scandinavian school-based programs for early identification and management of conditions like scoliosis, align with the principles of the CAS framework. These programmes adapt to adolescents’ needs fostering collaboration among schools, healthcare providers, and communities to enhance equity in MSK health outcomes, illustrating how CAS effectively captures the adaptive and interconnected nature of meso-level efforts, demonstrating their impact on macro-level goals and micro-level care.

 On a macro level, focusing solely on strengthening MSK health systems in low- and middle-income countries, driven by projected estimates in population aging, diverts attention from high-income countries that also bear a significant socioeconomic burden. MSDs affect individuals from lower socioeconomic backgrounds globally, irrespective of a country’s economic development, yet this aspect is not captured in the analysis. Ignoring the widespread nature of these issues undermines the effectiveness of health policies and neglects the full scope of the problem.

####  Funding

 The thematic analysis underscores the importance of person-centred care, prevention, early intervention, and integrated services, aligning with best practice models of care. Schneider and colleagues highlight a critical challenge: inadequate funding for research and innovation, necessary to drive whole-system reforms and support integrated healthcare. Despite the significant burden of MSDs, funding gaps prevent the implementation of these models, hindering the development of comprehensive MSK health policies and slowing progress in addressing the growing crisis.

 While the article advocates for robust data systems to monitor and evaluate services, such systems require substantial investment, which is often unavailable. This lack of funding results in insufficient data collection and analysis, creating gaps in evidence-based policy-making. Addressing these funding gaps is crucial, as highlighting the economic and social costs of MSDs could strengthen advocacy efforts and drive increased investment in MSK health. However, Schneider and colleagues stop short of proposing actionable solutions to address these funding challenges.

 They acknowledge broader gaps in global MSK health policies, including limited integration into wider health frameworks and a lack of strategies to address funding and implementation challenges. To bridge these gaps, actionable solutions such as collaboration with public health organizations and engagement with international funding bodies are needed. Partnerships with organizations like the WHO could support the development of region-specific policies, facilitating the implementation of integrated care models tailored to local needs. Collaborations with global funding organizations, such as the Global Fund or World Bank, could prioritize MSD within broader NCD strategies, providing resources for integrated care models, robust data systems, and capacity-building in underserved areas enabling a more equitable distribution of resources.

 Actionable steps should prioritize a life-course approach and address social determinants of health. Aligning MSK health initiatives with global priorities ensures policy effectiveness across diverse contexts. Integrating these strategies would enable Schneider and colleagues to transition from identifying gaps to proposing a comprehensive roadmap for strengthening global MSK health systems.

####  Integrated Care

 Nearly 50% of patients with MSDs experience comorbidities or multimorbidity, leading to increased polypharmacy, diminished quality of life, and higher healthcare utilisation, adding layers of complexity to the overall management of MSDs.^12^ The biopsychosocial impacts of MSDs, such as chronic pain, functional disability, work incapacity, psychological disorders, and increased mortality, increase the “wickedness” of this problem, underscoring the necessity for comprehensive, cross-sector integrated services addressing both the psychological and physical needs of patients.^13^ Without such an approach, transforming global MSK healthcare will remain an elusive goal, with health equity remaining out of reach.

 There is a move towards integrated healthcare models of which, the United Kingdom is a notable example. The United Kingdom introduced integrated care systems (ICS) in 2016, made statutory in 2022, to improve healthcare by coordinating care across sectors like the National Health Service, councils, and third-party organizations. These systems, through Integrated Care Boards, plan health services based on local needs. However, due to their early stage of development, there is limited understanding of how national MSK policies are effectively implemented in practice, particularly in addressing the growing public health issue of MSDs.

 Implementing ICS in diverse settings presents several challenges. First, varying healthcare infrastructure and resources hinder coordination, particularly in low- and middle-income countries. Second, weak data systems impede evidence-based planning and evaluation. Third, achieving cross-sector collaboration often requires overcoming cultural and operational silos, as well as addressing power dynamics among stakeholders. Finally, socioeconomic disparities and inequitable resource distribution remain significant barriers, especially in low-funded or unequal healthcare access areas. These challenges underscore the importance of tailoring ICS implementation to the specific needs and capacities of different regions, leveraging localized solutions while adhering to global best practices.

 As Schneider and colleagues suggest, the principles derived from their policy analysis can guide the formation of comprehensive, integrated MSK health policies at both national and global levels, ensuring that MSK health receives the attention and resources necessary for meaningful impact.

###  Policy Lagging Behind Practice

 The call for standardization aligns with the author’s findings, highlighting the persistent gap between policy and practice. Addressing this divide requires standardized tools, education, and resources to ensure consistent care quality across diverse settings. While clinical guidelines often reflect a reactive approach to patient needs, Schneider and colleagues’ work is significant in identifying this issue and advocating for solutions. Empowering healthcare providers with integrated, evidence-based care can be achieved through accessible guidelines, decision-making tools, and robust workforce education. These contributions add depth to the discussion and underscore the importance of translating policy into actionable strategies to advance MSK healthcare.

###  Lessons From Policy Implementation

 Effective health policy implementation hinges on robust monitoring, evaluation, and stakeholder engagement, yet limited research in this area has contributed to significant delays in translating policy into improved health outcomes. While Schneider and colleagues suggest further research into the history of the development MSK health policy to determine facilitating factors, this commentary advocates for evaluating the implementation of existing policies in countries with established national strategies. Such evaluations can yield valuable insights to guide the development of more effective global MSK policies.

 The authors highlight the context-dependent nature of policy learning, where lessons from one setting may not easily transfer to another. This underscores the need for standardized yet flexible implementation guidelines that accommodate local variations. By focusing on actionable steps and adaptable frameworks, policy-makers can create roadmaps for translating MSK health policies into practice.

 Applying complexity theory to this process underscores the need to navigate dynamic interactions, emergent behaviours, and feedback loops within health systems. A complexity-informed approach emphasizes interdependence, adaptability, and continuous learning, ensuring that MSK health policies not only address immediate needs but also evolve to tackle future challenges. This approach bridges the gap between policy design and real-world application, fostering equitable, sustainable, and impactful MSK healthcare worldwide.

## Conclusion

 This commentary has explored the current MSK health landscape and the challenges of translating policy into practice, leading to the following Wicked Question:

 “*How can MSK policy be strategically aligned and effectively implemented within integrated healthcare systems, considering the varying intensities of wickedness arising from systemic, socioeconomic, medical, and situational complexities?*”

 The complexity of the MSK burden is intensified by interwoven factors that complicate policy implementation, creating a “wicked problem,” where addressing MSK health in isolation is insufficient. The interconnectedness of these elements adds layers of complexity to the overall challenge, amplifying the wickedness of the issue.

 The wickedness of the MSK burden necessitates a shift from policy learning to actionable implementation. Regulatory reforms and health system restructuring present opportunities for a cross-sector, collaborative approach that integrates MSK policy through a life course perspective, offering the most promising solution. However, funding shortages remain a critical barrier to implementing the principles proposed by Schneider and colleagues. Bridging this gap will require increased investment, international cooperation, and integration into broader health strategies.

 Schneider and colleagues’ work has contributed significantly to this discourse, leading to the creation of the “Towards a Global Strategy to Improve Musculoskeletal Health” framework,^14^ which provides a structured, actionable approach to addressing the global MSK burden and fostering equitable, sustainable healthcare solutions on a global scale. However, the true challenge lies in addressing the complexity and wickedness of the issue, ensuring that these policies can be practically and effectively implemented across diverse contexts.

## Ethical issues

 Not applicable.

## Conflicts of interest

 Author declares that she has no conflicts of interest.
